# PhysioMio: bilateral and longitudinal HD-sEMG dataset of 16 hand gestures from 48 stroke patients

**DOI:** 10.1038/s41597-026-06557-0

**Published:** 2026-01-09

**Authors:** Julian Ilg, Alexander C. R. Oldemeier, Marie Fieweger, Luca Deuschel, Peter Rieckmann, Peter Young, Sabine Krause, Tim C. Lueth

**Affiliations:** 1 Institute of Micro Technology and Medical Device Technology (MIMED), Technical University Munich (TUM), Munich, Germany; 2 Medical Park Loipl, Bischofswiesen, Germany; 3 Medical Park Bad Feilnbach Reithofpark, Bad Feilnbach, Germany

**Keywords:** Stroke, Biomedical engineering, Electromyography - EMG

## Abstract

The PhysioMio dataset presented in this paper provides longitudinal high-density surface electromyography (HD-sEMG) recordings from both the healthy and impaired forearms of 48 stroke patients with arm paresis, captured during the performance of 16 distinct hand gestures. Patients were recorded at regular intervals during their individual inpatient rehabilitation stay, resulting in an average of three recording sessions at different stages of post-stroke rehabilitation per patient. The HD-sEMG signals were collected using a dry 64-electrode array positioned around the forearm, enabling observation of muscle activation patterns. The healthy arm was recorded during the first week to serve as a reference, while subsequent recordings focused solely on the impaired arm. This data can offer insights into neuromuscular deficits related to stroke and allow for comparative analysis between healthy and impaired arms. This dataset serves as a valuable resource for studying motor impairment and recovery potential in stroke-induced arm paresis, supporting advancements in personalized rehabilitation and assistive technologies.

## Background & Summary

With a prevalence of approximately 65% among stroke survivors, hemiparesis represents the most common motor impairment and constitutes a primary focus of treatment and rehabilitation in post-acute care^1^. In particular, the therapeutic management of arm paresis following stroke is considered a key element of stroke rehabilitation^2^. Impairments of the upper extremities, specifically affecting the movement and coordination of the arms, hands, and fingers, often result in significant difficulties in performing activities of daily living such as eating, dressing, and washing^3^. Consequently, the treatment of arm paresis has been identified by stroke survivors, caregivers and healthcare professionals as one of the top ten research priorities for life after stroke^4^.

Given the clinical relevance of upper arm impairments in stroke survivors, surface electromyography (sEMG) represents a promising tool to enhance rehabilitation strategies through objective, non-invasive, quantifiable insights into muscle activation patterns. To fully exploit this potential, comprehensive sEMG datasets from stroke patients are essential. Datasets capturing hand and finger movements are of critical importance, as fine motor skills are crucial for the performance of activities of daily living and are often severely affected after stroke. Such datasets enable the systematic analysis of neuromuscular function and facilitate the development of data-driven models for motor function assessment.

While several publicly available sEMG datasets focusing on hand and finger movements exist, the majority of these datasets have been collected from healthy individuals. An overview and comparison of the most relevant existing (HD-)sEMG datasets, including the few datasets that involve stroke survivors, is provided in Table 1.

**Table 1:** Comparison of PhysioMio dataset^5^ with other sEMG datasets for hand and finger movements.

Dataset	Subjects	Healthy/ Stroke	Gestures	EMG Channels	Longitudinal	Bilateral
Atzori *et al*.^7^	78	78/0	53	10/12	No	No
Di Domenico *et al*.^8^	10	10/0	16	64	No	No
Du *et al*.^9^	23	23/0	8/12	128	Yes	No
Gomez-Correa *et al*.^10^	28	28/0	10	4	No	No
Gowda *et al*.^11^	91	91/0	10	12	No	No
Guo *et al*.^12^	21	21/0	29	448	No	No
Israely *et al*.^13^	25	12/13	9	8	No	No
Jarque-Bou *et al*.^14^	22	22/0	26	7	No	No
Jiang *et al*.^15^	20	20/0	34	256	No	No
Kyranou *et al*.^16^	8	8/0	6	16	No	No
Malešević *et al*.^17^	20	20/0	65	128	No	No
Matran-Fernandez *et al*.^18^	25	25/0	13	134	No	No
Pradhan *et al*.^6^	43	43/0	16	28	Yes	No
Zhao *et al*.^19^	40	20/20	35	10	No	No
**PhysioMio dataset**^5^	**48**	**0/48**	**16**	**64**	**Yes**	**Yes**

To address the lack of large-scale, high-quality HD-sEMG datasets in the context of stroke rehabilitation, we conducted a clinical study involving 48 stroke survivors. To the best of our knowledge, the resulting PhysioMio dataset^5^ represents the largest collection of HD-sEMG recordings focused on hand and finger movements in stroke patients to date. HD-sEMG was employed to capture detailed neuromuscular activity across 64 electrodes, enabling a high spatial resolution of muscle activation patterns. In contrast to many existing datasets that are limited to isolated snapshots, our recordings encompass continuous four-second segments of muscle activity for each gesture, thereby facilitating time-resolved analyses of neuromuscular function. In addition, patients were recorded throughout their individual inpatient rehabilitation stay to monitor potential longitudinal changes in muscle activity during the course of recovery. Furthermore, bilateral recordings from both the paretic and non-paretic sides were acquired systematically, allowing direct intra-subject comparisons of neuromuscular function for each gesture.

This dataset offers considerable potential for reuse across a range of research applications, including, but not limited to, the development and validation of machine learning models for movement classification, the investigation of compensatory motor strategies following stroke and the advancement of personalized rehabilitation approaches.

## Methods

### Study participants

Fourty-eight stroke patients with hemiparesis (20 female, 28 male) participated in the study. Of these, 44 were right-handed and 4 were left-handed. In 26 patients, the left arm was paretic and in 22 the right, while the dominant arm was affected in 22 patients and the non-dominant arm in 26. The median age of the participants was 69 years (±13.8), ranging from 25 to 90 years. The median body weight was 75.5 kg (±12.3), with a range of 58 to 101 kg. The median height was 170.5 cm (±8.2), ranging from 157 to 190 cm. The median time post-stroke at the time of testing was 35.5 days, ranging from 13 to 2,308 days. One patient was classified as having chronic hemiparesis (more than six months post-stroke) and represented an outlier in the time since stroke, with a duration of 2,308 days. Each subject participated in a median of six recording sessions (range: three to fourteen), conducted throughout their inpatient rehabilitation stay. A total of 92 assessments were conducted on the healthy arm and 237 on the impaired arm of the stroke patients. Table 2 gives an overview of assessments included in the dataset.

**Table 2:** Metadata of the recruited stroke patients for the PhysioMio dataset^5^ (SD = standard deviation).

Characteristic	PhysioMio dataset^5^
Gender (m/f)	20/28
Paretic arm (l/r)	26/22
Dominant arm (l/r)	4/44
Age (y)	Median: 69.0; SD: ± 13.8; Range: 25–90
Weight (kg)	Median: 75.5; SD: ± 12.3; Range: 58–101
Height (cm)	Median: 170.5; SD: ± 8.2; Range: 157–190
Time post stroke (days)	Median: 35.5; Range: 13–2,308
Executed assessments per patient (number)	Median: 6; SD: ± 2.9; Range: 3–14
Total assessments healthy arm (number)	92
Total assessments impaired arm (number)	237

The study was approved by the ethics committee at Technical University Munich (2023-273-S-KH) according to the Declaration of Helsinki. The study was registered at the German Clinical Trials Register (DRKS) with the ID DRKS00032380. Each participant was briefed by a medical doctor who informed the participants about the procedure and potential risks before engaging in the study. Each participant was given a patient information sheet and a declaration of consent for the study prior to their participation where they consented that the acquired health data can be used and published for medical research. Patients were excluded from the study if they had severe physical or neurological impairments that prevented measurement, were unable to understand the instructions, or were unable to give consent. The HD-EMG data and metadata were stored locally on an encrypted hard drive in pseudonymized form. Before transferring the data to the hard drive, each patient was assigned a pseudonym in the form of a randomly generated Universal Unique Identifier, which links the HD-EMG data and metadata to the patient. The pseudonym–real name assignment was kept offline on a separate encrypted USB stick and was deleted after the end of the study.

### Instrumentation and materials

**Fig. 1:**
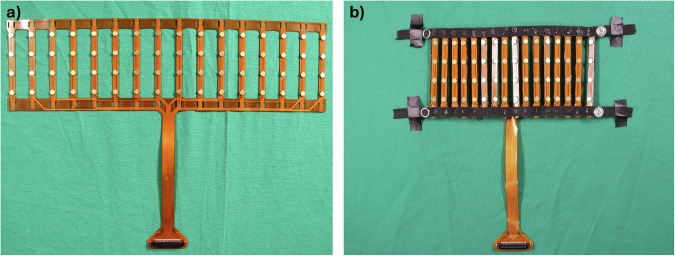
HD-sEMG electrode array used for the data acquisition: (**a**) before assembly and (**b**) with stretchable straps to adjust to different arm circumferences.

HD-sEMG signals were acquired in monopolar acquisition mode using the OT Bioelettronica Quattrocento (OT Bioelettronica, Turin, Italy). The Quattrocento is a commercial, Medical Device Regulation (MDR) 2017/745-certified EMG amplifier, that was used with a sampling frequency of 2048 Hz for all HD-sEMG signal acquisitions in this study. A 64-channel, custom-designed, hybrid rigid-flexible electrode array with goldplated, dry electrodes provided by OT Bioelettronica (see Fig. 1a)) was used for acquiring the HD-sEMG signals on the skin. This electrode array consists of 16 columns, each with four electrodes equidistantly spaced on a rigid Printed Circuit Board (PCB) stripe. These stripes are connected by flexible PCBs to ensure easy use on measurement areas and subjects of differing sizes. To attach the electrodes to the subjects’ forearms, two elastic bands were fixed to each end of the rigid columns under the connection to the flexible PCB (see Fig. 1b)). Snap buttons were used as a fixing mechanism. This ensured a tight, proper fit of the HD-sEMG electrodes on patients with differing circumferences of the lower arm.

**Fig. 2:**
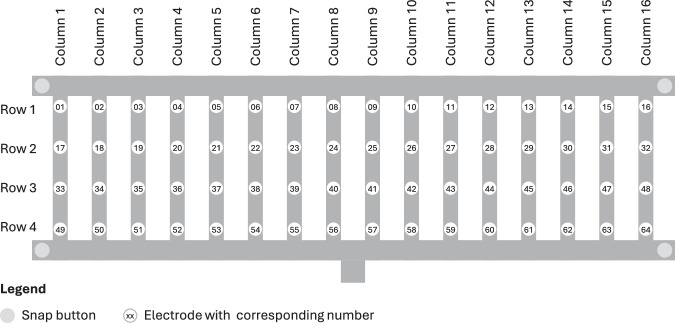
Layout and numbering of the 64 electrodes in the HD-sEMG array, shown with the electrodes oriented upwards towards the viewer.

To facilitate a clear interpretation and assignment of the recorded signals, the electrodes of the array were systematically numbered from 01 (row 1 and column 1) to 64 (row 4 and column 16), as shown in Fig. 2.

OT BioLab + (OT Bioelettronica, Turin, Italy) was used as software for the acquisition of the HD-sEMG data. The software saves the data to a proprietary data type.otb + , which can be exported to typical data types in data analysis, like Comma-Separated Values (CSV). Furthermore, it enables a live visualization of the data to check for interference and other noise.

In addition to the acquisition of HD-sEMG data, the Quattrocento system was also configured to collect a trigger signal to label the start and the end of the data acquisition for each movement. To facilitate this, a foot trigger mechanism was implemented between a USB plug and a BNC connector. Both connectors were manually soldered together, allowing for the transfer of a 5 V signal from the USB to the BNC. All the trials were recorded in a video to be able to check the execution of the respective movements to ensure proper data validation. The videos are not included in the PhysioMio dataset^5^ to ensure the subjects’ privacy.

**Fig. 3:**
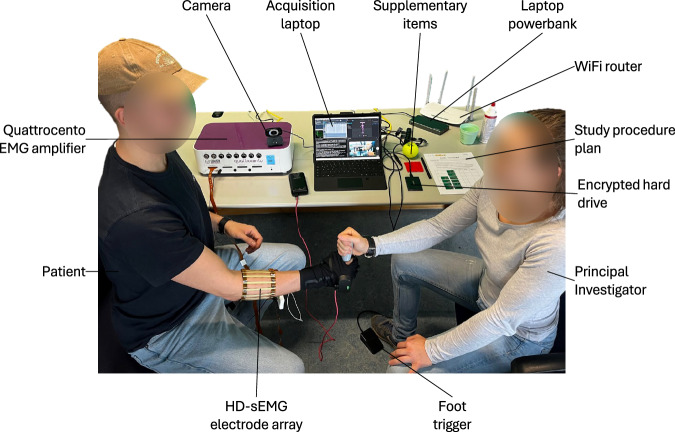
Overview of clinical study setup for data acquisition.

The data was acquired on a *Microsoft* Surface Pro 8 (Core i7-1185G7, 16GB RAM, 256GB SSD) (Microsoft Corporation, 2021) running Windows 11 Professional using a custom Python module, controlled via a command line interface. This module is part of a custom package ensuring standardized, uniform data acquisition and storage of the HD-sEMG data. It ensured synchronous acquisition of video and HD-sEMG data and stores the data on an encrypted, external hard drive. During data acquisition, the power plug was disconnected from the computer to mitigate powerline interference. Instead, a laptop power bank was used, which supplied the laptop with direct current (DC) directly. This way no alternating current (AC) was introduced by the charging system. Figure 3 shows the experimental setup of the study.

### Acquisition protocol

Data was collected over a period of eight months at two stroke rehabilitation clinics. Each patient was recorded longitudinally throughout their individual inpatient rehabilitation stay. Since the length of stay varied across patients, the number of recording sessions differs from patient to patient. Prior to each recording session, all systems were tested according to the manufacturer’s instructions to ensure proper functionality.

**Fig. 4:**
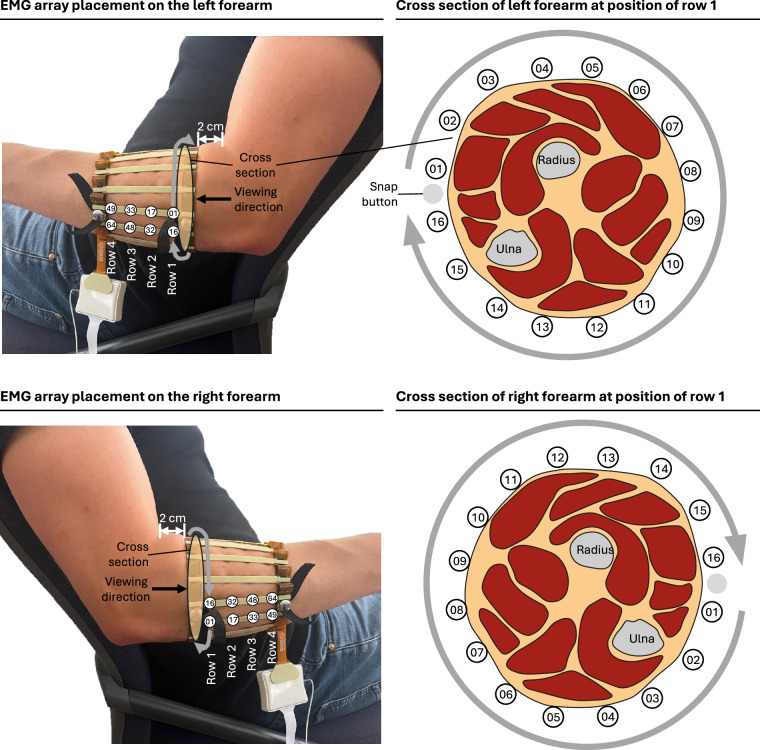
Position of HD-sEMG electrode array on respective forearms.

The subject’s skin on the lower arm and the electrode array were disinfected with alcohol and the electrode array was positioned 2 cm from the patient’s crook of the arm and tightly wrapped around the lower arm to ensure proper contact between the electrodes and the skin. The array was aligned such that, during recordings on the left forearm, the electrode column 15 (electrodes 15, 31, 47 and 63) was positioned over the ulna, with the connection cable oriented towards the subject’s hand. For recordings on the right forearm, the electrode column 2 (electrodes 2, 18, 34 and 50) was positioned over the ulna as well, with the cable likewise oriented towards the hand (see Fig. 4).

The first session of a subject consisted of up to four assessments in total, one to two assessments were dedicated to recording baseline reference data from the healthy arm, and one to two assessments were recorded from the impaired arm. In all subsequent sessions, only the impaired arm was recorded with one to two assessments per session. Variations in the number of assessments were primarily due to patient fatigue, which is an expected factor in clinical studies.

**Fig. 5:**
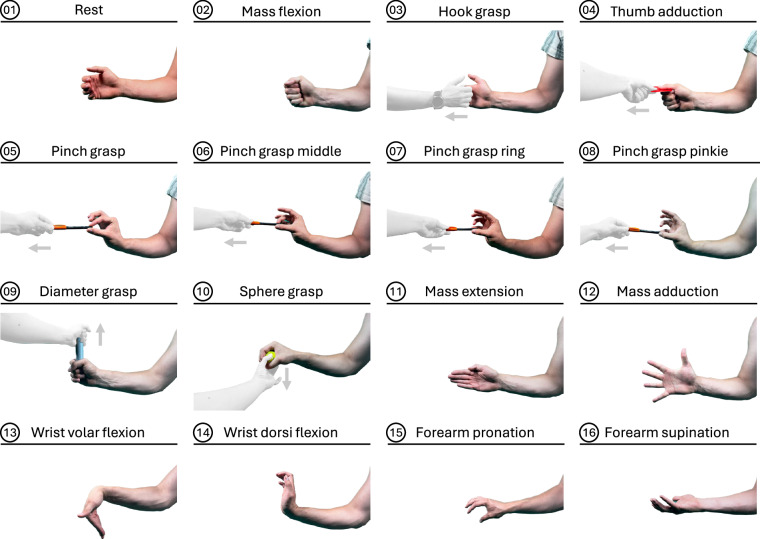
Gesture set with 16 distinct gestures for the study; gestures 3–10 involve an interaction with the PI who tries to pull with moderate force towards the indicated direction (light grey).

In each assessment, the subject had to perform 16 gestures. The set of gestures included one resting pose to capture baseline signals with little muscle activity, along with 15 other distinct gestures, as illustrated in Fig. 5. Some gestures required additional items and interaction with the principal investigator (PI). The patients were motivated to execute or interact as strongly as possible throughout all performed gestures. According to the definition of the Fugl-Meyer Assessment (FMA), the executed movements were rated from the PI on a three-point ordinal scale ranging from 0 to 2. A score of 0 indicated that the movement could not be performed, 1 that the movement could be partially performed, or the limb could not maintain position against resistance, and 2 that the movement could be fully performed or sustained against resistance. The recording of each assessment took approximately 10–15 minutes and was followed by a short break to allow patients to recover.

Prior to each gesture, the PI explained and demonstrated the gesture to the subject. The subject repeated the gesture and as soon as the subject reached the correct pose, the PI started counting for seven seconds. The PI pressed a foot trigger after the first second and released it after six seconds to ensure sufficiently long timing and to prevent premature release by the participant. If the subject was unable to fully execute the required gesture, the counting was initiated from the maximum position the subject was able to attain.

Throughout the procedure, the PI monitored the execution and recording of each gesture. If necessary, data collection of certain gestures was repeated to ensure accuracy. The PI assessed each movement via the FMA scale. All attempts were included in the PhysioMio dataset^5^. If a patient was unable to perform the gesture, the trial was retained and assigned an FMA score of 0. After completion of all recorded assessments, the electrodes were removed from the subject and the subject’s forearm as well as all objects used during the session were disinfected.

### Data processing

The HD-sEMG data for each recording were stored as continuous recordings lasting approximately 10 to 15 minutes, comprising signals from all 64 electrodes during the performance of 16 distinct hand gestures as well as the intervening transition periods.

During signal acquisition, a digital high-pass filter at 10 Hz and a digital low-pass filter at 500 Hz, configured via the OT BioLab + (OT Bioelettronica, Turin, Italy) software, were applied to the recorded data. Notably, no notch filter was applied to suppress potential power line interference. Users intending to apply a notch filter should consider that the recordings were made in Germany, where the mains frequency is 50 Hz.

A trigger signal marking the onset and offset of each gesture was synchronously recorded alongside the HD-sEMG signals and was digitally debounced during post-processing.

The recordings and trigger signals were processed using OT BioLab + and initially saved in the proprietary otb + file format. To ensure compatibility with a broader range of analysis tools, the data were exported to comma-separated values (CSV) format and then converted to Apache Parquet format using Python, enabling more efficient storage and faster data processing.

Gesture segmentation was performed based on synchronized trigger signals. Since the initiation and termination of each gesture were manually controlled by the PI, slight variations in duration occurred. To standardize the PhysioMio dataset^5^, the central four seconds of each labeled gesture were extracted, ensuring uniform segment lengths across all recordings. Transition periods between gestures were excluded from the final PhysioMio dataset^5^.

Each assessment was saved as a separate Parquet file named XX.parquet, where XX represents the respective recording number. Each file contains 66 columns. The “time” column represents the timestamp in seconds relative to the start of the individual gesture. Columns “channel_01” to “channel_64” contain the raw HD-sEMG signals from the 64 electrodes, expressed in mV and numbered as shown in Fig. 2. The column “fma” shows the evaluation of the respective gesture. For rest poses the “fma” field is empty. The “movement_type” column indicates the classified gesture, corresponding to a four-second segment for each gesture.

## Data Records

**Fig. 6:**
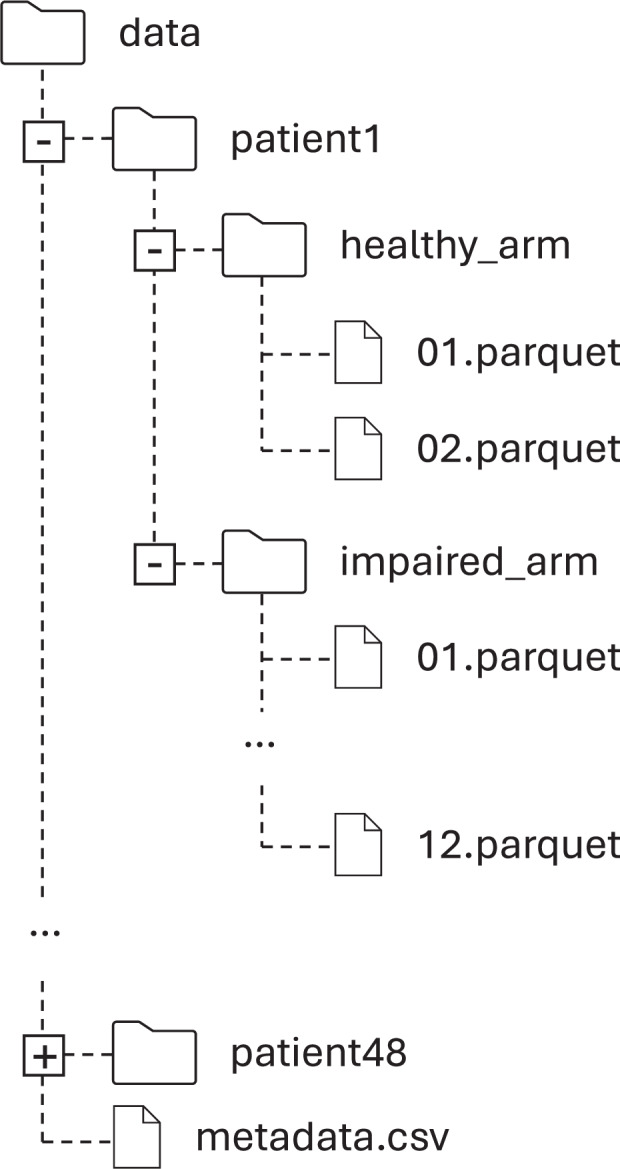
Structure of the PhysioMio dataset^5^ available at Hugging Face.

The PhysioMio dataset collected in the study can be downloaded from Hugging Face^5^. The data is organized according to the file structure illustrated in Fig. 6.

For each participant, a dedicated folder named sequentially from *patient1* to *patient48* is provided, containing all corresponding data. Within each participant folder, two subfolders are available: one for the recordings from the healthy arm and one for those from the impaired arm.

The *healthy_arm* folder contains up to two Parquet files, each representing one assessment of the healthy arm acquired during the initial recording session.

The *impaired_arm* folder contains up to twelve Parquet files, with the number corresponding to the number of recorded assessments conducted for the impaired arm.

A metadata file in csv-format accompanies the PhysioMio dataset^5^ to provide participant information and contextualize the recordings. All metadata is fully anonymized and does not contain any personally identifiable information. Each row corresponds to one participant and includes the following columns:

- **id:** A unique participant identifier, ranging from *patient01* to *patient48*.
- **arm_type:** Arm type for each recording, recorded as *healthy_arm* or *impaired_arm*.
- **recording_index:** The number of the respective recorded assessments per patient and arm type, starting with 1.
- **file_path:** The file path of the recording relative to the data folder.
- **sha256sum:** Checksum of the file.
- **age_in_years:** Participant’s age in years at the time of the first recording.
- **gender:** Participant’s gender, recorded as *m* for male or *f* for female.
- **height_in_cm:** Participant’s height in centimeters at the time of the first recording.
- **weight_in_kg:** Participant’s weight in kilograms at the time of the first recording.
- **impaired_arm:** Participant’s side of the body affected by the stroke, recorded as *l* for left and *r* for right.
- **dominant_arm:** Participant’s dominant arm prior to the stroke, recorded as *l* for left and *r* for right.
- **days_after_stroke:** Number of days after the stroke event at which the recording was performed.

Standard participant information, including gender, weight, and height, is provided in the metadata file. Additionally, the information on the number of days after stroke for each recording allows users to reconstruct the longitudinal course of data acquisition for each participant. The PhysioMio dataset^5^ contains 329 files and has a size of 4,42 GB.

## Technical Validation

The EMG system was applied and used according to the manufacturer’s specifications to ensure accurate and reproducible signal acquisition. Prior to each recording session, a test contraction was performed to verify signal quality. In cases where elevated baseline noise was detected, an error protocol was followed to identify and address potential sources of interference (e.g., poor skin contact, cable motion artifacts).

After processing the data, we conducted a secondary filtering step by manually reviewing every recording through an intuitive visualization interface, allowing rapid identification and removal of recordings with strong residual artifacts.

To further validate the final PhysioMio dataset^5^, we applied quantitative signal quality metrics, including signal-to-noise ratio (SNR), coefficient of correlation to a normal distribution (CCN), power spectral density (PSD), and a basic classifier to separate healthy and impaired patients.

### Secondary filtering

We conducted an additional manual quality-control pass to ensure the high quality of the final PhysioMio dataset^5^. For each recording, we generated a compact visual summary that displayed the time-series traces of all channels alongside channel statistics over the SNRs of all movements in this channel. SNRs were computed in decibel (dB) treating the *Rest* gesture as noise $N$ and each of the 15 remaining movements ${m}_{i}$ as separate signals:

$${SN}{R}_{{m}_{i}}=20\,{\log }_{10}\left(\frac{{mean}(||{m}_{{ij}}||)}{{mean}(||{N}_{j}||)}\right)$$

**Fig. 7:**
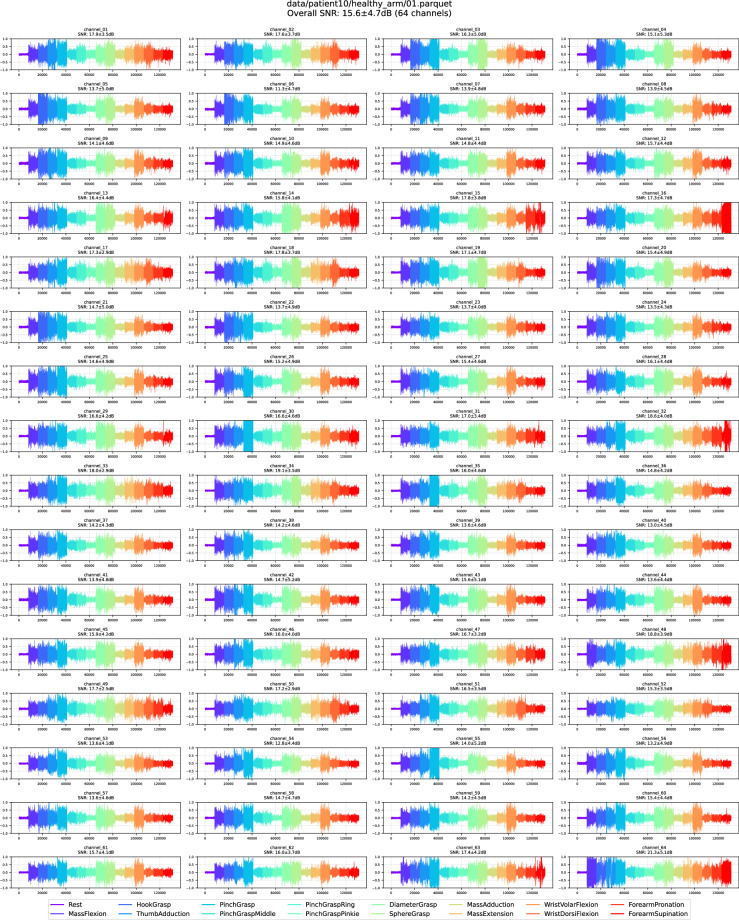
Retained recording of a healthy arm showing the HD-sEMG signals across all 64 channels. The different colors indicate the different performed gestures in consecutive 4-second segments.

**Fig. 8:**
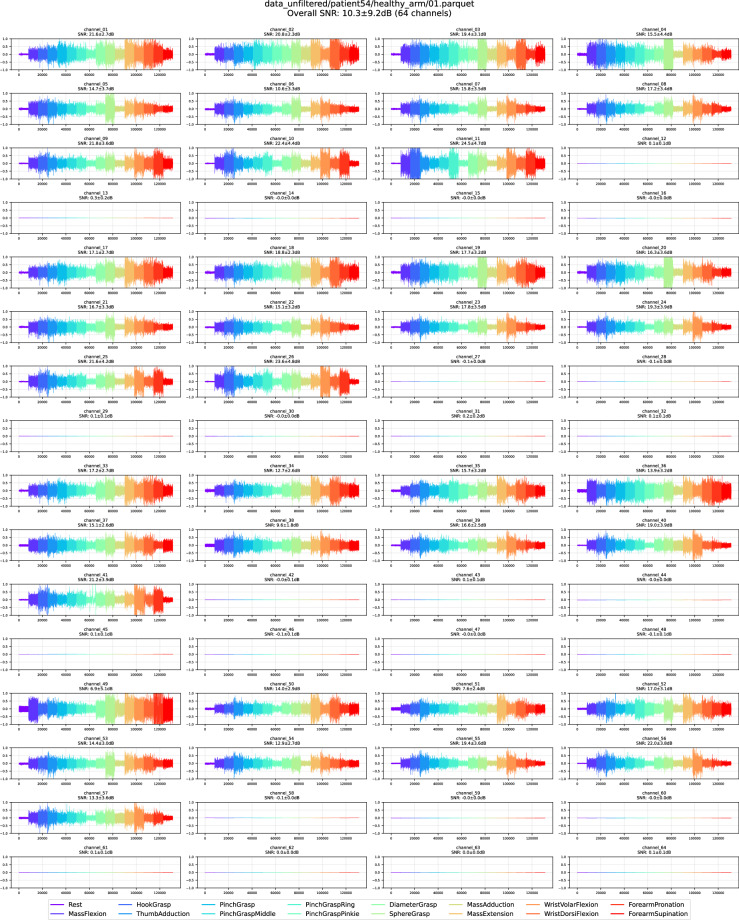
Discarded recording of a healthy arm showing the HD-sEMG signals across all 64 channels with a high number of malfunctioning channels. The different colors indicate the different performed gestures in consecutive 4-second segments.

For each recording, we generated an overall SNR statistic (SNR and standard deviation) by pooling the SNRs for all movements for all 64 channels. Recordings exhibiting uniformly low SNRs (<2.5 dB), SNRs with high standard deviation, or unusually large inter-movement SNR were flagged, and their traces were inspected visually and excluded from the final PhysioMio dataset^5^. If more than 10% of the 64 channels for a respective movement did not record an HD-sEMG signal due to missing skin contact, the entire recording was excluded. The two panels below illustrate this procedure: Fig. 7 shows a recording retained in the PhysioMio dataset^5^, whereas Fig. 8 with its conspicuous channel failure was discarded, resulting in the removal of the associated patient from the final PhysioMio dataset^5^.

This screening step resulted in the exclusion of six patients and 45 individual recordings from the final PhysioMio dataset^5^. These were removed prior to the statistical summary presented at the beginning of this paper (as shown in Table 2), which reflects the final, quality-controlled PhysioMio dataset^5^. A small number of recordings still exhibit sporadic channel dropouts or elevated noise levels. Such artefacts are inevitable when using a 64-electrode array, especially during active tasks performed with a paretic arm.

### Healthy vs. Impaired arm

**Fig. 9:**
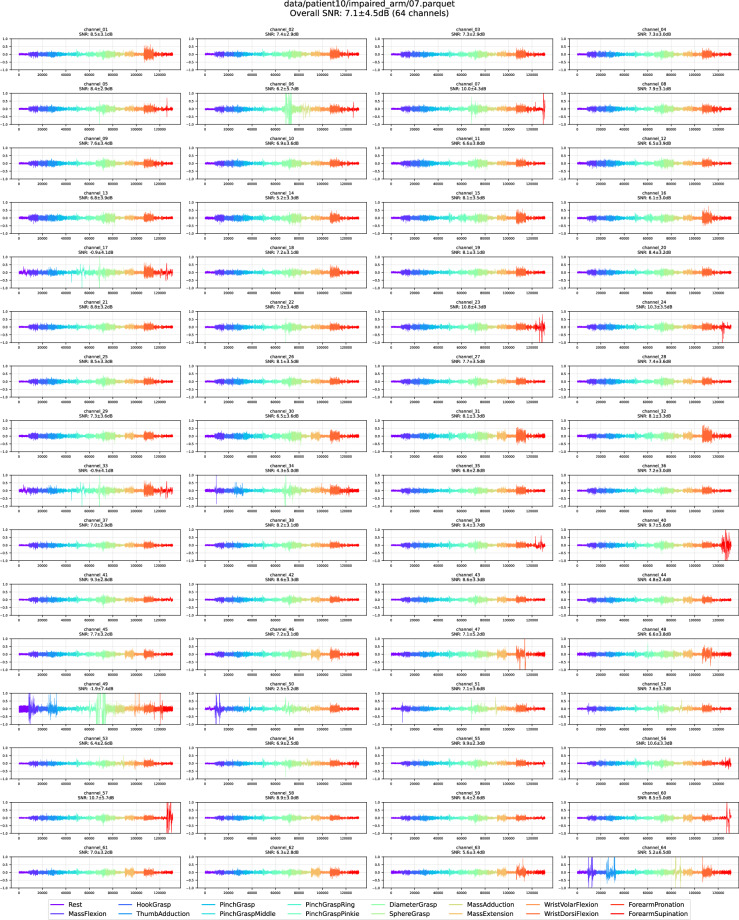
Recording of an impaired arm which illustrates the lower overall HD-sEMG amplitude across all 64 channels. The different colors indicate the different performed gestures in consecutive 4-second segments.

Recordings from neurologically intact arms differ markedly from those obtained from paretic arms, especially in cases of pronounced paresis. In Fig. 9, for example, the channel amplitudes are substantially attenuated in comparison to Fig. 7, producing a markedly lower signal-to-noise ratio (SNR). Moreover, compensatory movements required to perform the required gesture can in some cases introduce additional motion artefacts, which can affect the SNR.

### Signal-to-noise ratio (SNR)

For the curated PhysioMio dataset^5^, we computed patient-level SNR statistics (SNR and standard deviation) by pooling SNR for each movement for all channels for each recording separately for healthy and impaired arms. As an overall statistic we provide the means of SNR and standard deviation over all patients separately for healthy and impaired arms. All results are summarized in Table 3.

**Table 3:** Signal-to-noise ratio (SNR) and standard deviation (SD) across all patients included in the PhysioMio dataset^5^, separated by healthy vs. impaired arm.

Patient	SNR of healthy arm [dB]	SNR of impaired arm [dB]
Patient 1	17.86 ± 6.96	11.79 ± 5.81
Patient 2	15.93 ± 6.31	6.69 ± 5.70
Patient 3	21.01 ± 6.04	12.14 ± 5.73
Patient 4	16.22 ± 5.15	12.14 ± 5.42
Patient 5	21.02 ± 7.02	11.74 ± 3.74
Patient 6	10.69 ± 4.05	8.54 ± 4.26
Patient 7	10.72 ± 4.86	7.43 ± 4.56
Patient 8	14.25 ± 4.03	11.07 ± 4.39
Patient 9	18.43 ± 8.62	12.19 ± 5.77
Patient 10	14.62 ± 4.51	11.19 ± 4.67
Patient 11	13.52 ± 5.51	7.73 ± 8.16
Patient 12	10.18 ± 3.36	7.88 ± 5.84
Patient 13	5.43 ± 3.48	4.62 ± 5.03
Patient 14	8.50 ± 5.05	5.96 ± 4.63
Patient 15	5.73 ± 2.97	15.63 ± 5.36
Patient 16	7.46 ± 5.86	10.12 ± 5.88
Patient 17	9.73 ± 4.67	10.42 ± 5.45
Patient 18	13.82 ± 4.17	5.02 ± 3.64
Patient 19	12.30 ± 6.34	5.01 ± 4.71
Patient 20	14.63 ± 6.04	9.85 ± 5.85
Patient 21	17.65 ± 8.31	17.62 ± 7.86
Patient 22	17.52 ± 6.25	11.74 ± 5.03
Patient 23	17.70 ± 7.14	14.83 ± 6.81
Patient 24	13.55 ± 4.84	6.19 ± 5.52
Patient 25	17.60 ± 5.08	12.28 ± 5.46
Patient 26	14.86 ± 3.72	10.26 ± 5.33
Patient 27	11.06 ± 7.42	10.96 ± 5.71
Patient 28	14.02 ± 5.19	6.93 ± 4.50
Patient 29	12.85 ± 4.46	10.82 ± 4.85
Patient 30	14.33 ± 6.39	13.78 ± 5.38
Patient 31	9.18 ± 5.60	10.18 ± 5.73
Patient 32	21.92 ± 7.28	19.71 ± 5.92
Patient 33	12.43 ± 6.43	13.28 ± 6.67
Patient 34	12.90 ± 5.71	11.46 ± 4.95
Patient 35	9.63 ± 3.83	7.22 ± 4.87
Patient 36	13.48 ± 4.35	5.38 ± 2.67
Patient 37	11.00 ± 5.80	15.10 ± 6.28
Patient 38	18.80 ± 8.23	15.92 ± 7.88
Patient 39	10.29 ± 7.39	11.74 ± 6.18
Patient 40	12.86 ± 6.67	15.07 ± 6.41
Patient 41	8.79 ± 5.05	5.71 ± 5.61
Patient 42	10.70 ± 4.47	9.19 ± 5.11
Patient 43	10.30 ± 5.17	5.77 ± 4.68
Patient 44	13.82 ± 6.70	12.84 ± 5.70
Patient 45	13.08 ± 5.34	7.58 ± 4.00
Patient 46	17.34 ± 3.87	11.10 ± 4.95
Patient 47	14.96 ± 4.76	5.31 ± 4.82
Patient 48	12.86 ± 4.85	5.36 ± 4.58
**Means across patients**	**13.49** ± **5.53**	**10.22** ± **5.38**

The results presented above clearly demonstrate the presence of a meaningful signal during gesture execution compared to baseline resting conditions. Applying the same methodology to the initial subset of the Ninapro dataset (DB1) yields an average SNR of 17.90 ± 6.84 dB. It should be noted, however, that these values correspond to young, healthy subjects, and thus serve primarily as a reference for optimal physiological conditions.

### Correlation coefficient of Normality (CCN)

A normally distributed signal amplitude is a sign for a good EMG signal, whereas a signal amplitude with a non-normal distribution would be considered contaminated^6^. We quantify how closely our signal amplitudes match a normal distribution by calculating the *Correlation Coefficient of Normality* (CCN), which is defined as the Pearson correlation between the empirical amplitude histogram and an ideal Gaussian distribution having identical mean and variance:

$${CCN}=r\left({p}_{{empirical}}\left(a\right),{p}_{N\left(\mu ,{\sigma }^{2}\right)}\left(a\right)\right)$$

A CCN value approaching 1 denotes a near-Gaussian amplitude distribution, which is indicative of a clean EMG trace. We aggregated CCN values across all retained recordings and report pooled summary statistics separately for healthy and paretic arms (see Table 4).

**Table 4:** Pooled Correlation coefficient of Normality (CCN) statistics for the healthy and impaired arms of the PhysioMio dataset^5^.

Type	Mean CCN	Standard deviation
Healthy arm	0.896	0.087
Impaired arm	0.904	0.079

The PhysioMio dataset^5^ achieves a robust CCN of 0.896 ± 0.087 for the healthy arm, which is comparable to the CCN of the widely used Ninapro dataset (0.848 ± 0.075)^6^.

### Spectral analysis

To characterize the frequency content of the HD-sEMG, we estimated the power-spectral density (PSD). Each channel was first high-pass filtered with a fourth-order Butterworth filter (cut-off = 20 Hz) to suppress motion artefacts and then filtered at 50 Hz with a second-order notch filter to remove mains interference. PSDs were obtained via Welch’s method using Hann windows of 1024 samples with 50% overlap.

**Fig. 10:**
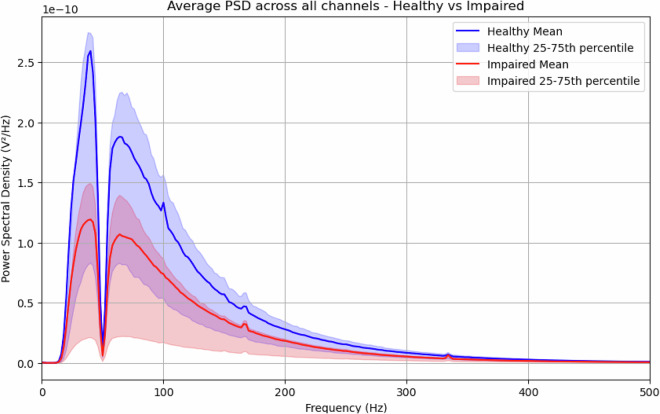
Power-spectral density analysis of the PhysioMio dataset^5^. The blue line represents the mean PSD for the recordings of the healthy arm while the lower and upper border of the shaded area represent the 25^th^ and 75^th^ percentile respectively. The red line represents the mean PSD for the recordings of the impaired arm with the same shaded area to represent the 25^th^ and 75^th^ percentile.

The resulting spectra in Fig. 10 displays the canonical EMG power band (≈20–500 Hz). Although healthy and paretic arms share peak frequencies, the impaired recordings consistently exhibit a lower overall spectral magnitude, consistent with the reduced motor-unit recruitment expected in severe paresis.

### Basic classifier to separate healthy and impaired arms

To complement the signal-quality analyses, we assessed whether a basic classifier could discriminate between recordings from the healthy and paretic arms. Given the significant differences observed in these features (see Table 3 and Fig. 10), the per-recording PSD (20 frequency bins) together with the SNR mean and standard deviation were merged into a feature matrix. Each feature was normalized per patient using min–max normalization to mitigate inter-subject variability. Figure 10 suggests that the main difference between healthy and impaired PSD curves is a linear factor, so we simplified the feature space further by selecting only two normalized PSD frequency bands (68–92 Hz, 236–260 Hz), resulting in four final features. A Random Forest classifier (with fixed hyperparameters) was then trained using patient-based data splits to prevent subject leakage.

In a held-out evaluation with patients entirely unseen during training, the model achieved Accuracy = 0.878, Balanced Accuracy = 0.877, Macro-F1 = 0.865, AUROC = 0.890, and AUPRC = 0.913. A 5-fold patient-based cross-validation yielded 0.855 with per fold accuracy of 0.840, 0.924, 0.955, 0.677 and 0.878 and balanced accuracy of 0.859 with per fold balanced accuracy of 0.855, 0.949, 0.963, 0.641 and 0.888. A stricter leave-one-patient-out (LOPO) evaluation gave 0.851 micro-accuracy and 0.874 macro-accuracy (averaged across per-patient results) with 0.856 micro-balanced accuracy and 0.870 macro-balanced accuracy.

These consistent results demonstrate that even a simple, out-of-the-box model can separate healthy from impaired recordings, corroborating the PhysioMio dataset’s^5^ validity and the systematic signal differences between arms observed in our SNR and spectral analyses.

The PhysioMio dataset^5^ on its own is inherently imbalanced for this task, due to recording the healthy arm only once or twice per patient during the initial sessions, while the impaired arm was recorded longitudinally throughout rehabilitation (≈1: 3.5 ratio). This was addressed in two ways. Firstly, all splits were patient-wise, eliminating data leakage and ensuring balanced contribution of each subject. Secondly, the Random Forest was trained with class_weight = ‘balanced’, ensuring equal effective weighting of both classes. The close agreement between balanced accuracy and overall accuracy, together with high AUPRC, indicates that the classifier did not exploit the imbalance spuriously and that both classes were learned effectively.

This classification experiment serves only as a baseline validation, deeper investigation into features, model architecture and combining it with other datasets are interesting topics for future research based on the PhysioMio dataset^5^.

## Usage Notes

The PhysioMio dataset^5^ is hosted in a public Hugging Face repository. The authors of this paper consented to the sharing of the data.

The users are advised to preprocess the PhysioMio dataset^5^ upon receiving the data. This includes filtering the desired EMG power band (e.g., 20–500 Hz) and applying a notch filter at 50 Hz to suppress potential power line interference. Please see the next section for example code.

## Data Availability

The presented PhysioMio dataset can be downloaded from Hugging Face (10.57967/hf/6783)^5^.

## Code availability

The code required to preprocess the PhysioMio dataset^5^ and to reproduce the analyses above is available at the PhysioMio GitHub repository https://github.com/formove-ai/physiomio. The scripts were developed and written in Python 3.11 with standard open-source software packages such as matplotlib, numpy, and scikit-learn.
